# Supplementary material for: Meta-Analysis on the Global Prevalence of Tetracycline Resistance in Escherichia coli Isolated from Beef Cattle
Source: Vet Sci. 2023 Jul 21;10(7):479. doi: 10.3390/vetsci10070479 (PMC10385540; doi:10.3390/vetsci10070479)
Supplement: Supplementary file 1 [file vetsci-10-00479-s001.zip › vetsci-2453365-supplementary.pdf]

veterinary sciences

MDPI

Supplementary Materials

Meta-Analysis on the Global Prevalence of Tetracycline Resistance in *Escherichia coli* Isolated from Beef Cattle

Yohannes E. Messele, Gebremeskel Mamu Werid and Kiro Petrovski

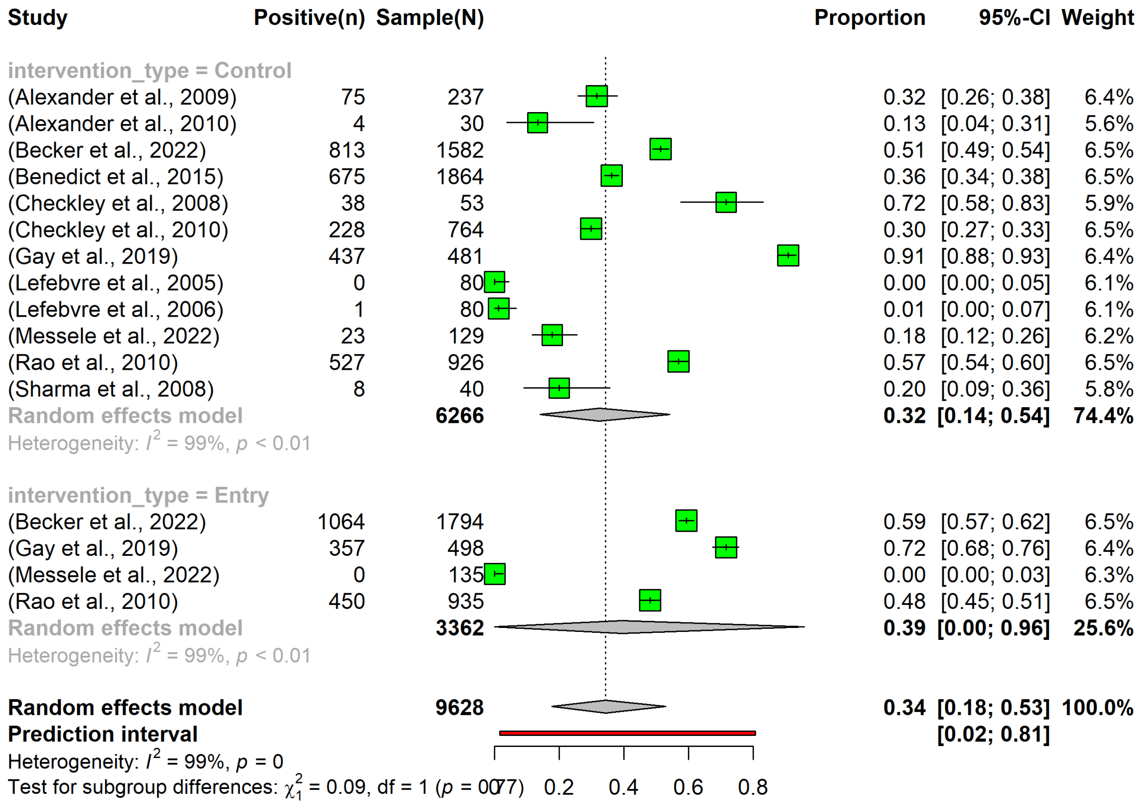

Supplementary Figure S1. Prevalence of tetracycline resistance in Escherichia coli isolates obtained from beef cattle without any antibiotic intervention, after 'Alexander et al., 2008'[26] and 'Benedict et al., 2015'[32] were removed as outliers. Samples were collected upon the entry of cattle into the feedlots ('Entry') and at the time of their exit ('Control'). It's important to note that some of the studies classified under the 'Control' subgroup were part of case-control studies, while others originated from cohort observational studies [4, 24, 25, 27–31, 33–36].

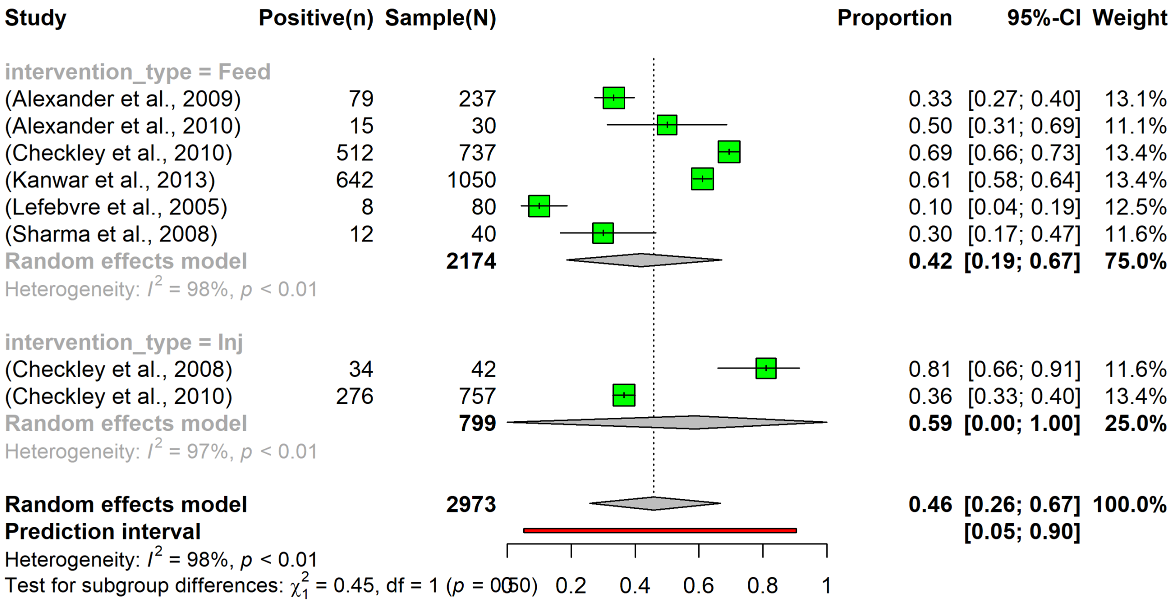

Supplementary Figure S2. Prevalence of tetracycline resistance in Escherichia coli isolates sourced from beef cattle subjected to antibiotic intervention After 'Alexander et al., 2008' [26]and 'Lefebvre et al., 2006'[34] were removed as outliers [24, 25, 27–30, 37]

The tetracycline was administered sub-therapeutically via either feed (indicated as 'Feed') or an injection (denoted as 'InJ'). To examine the dynamics of tetracycline resistance following an intervention, samples were collected at various time points post-administration.

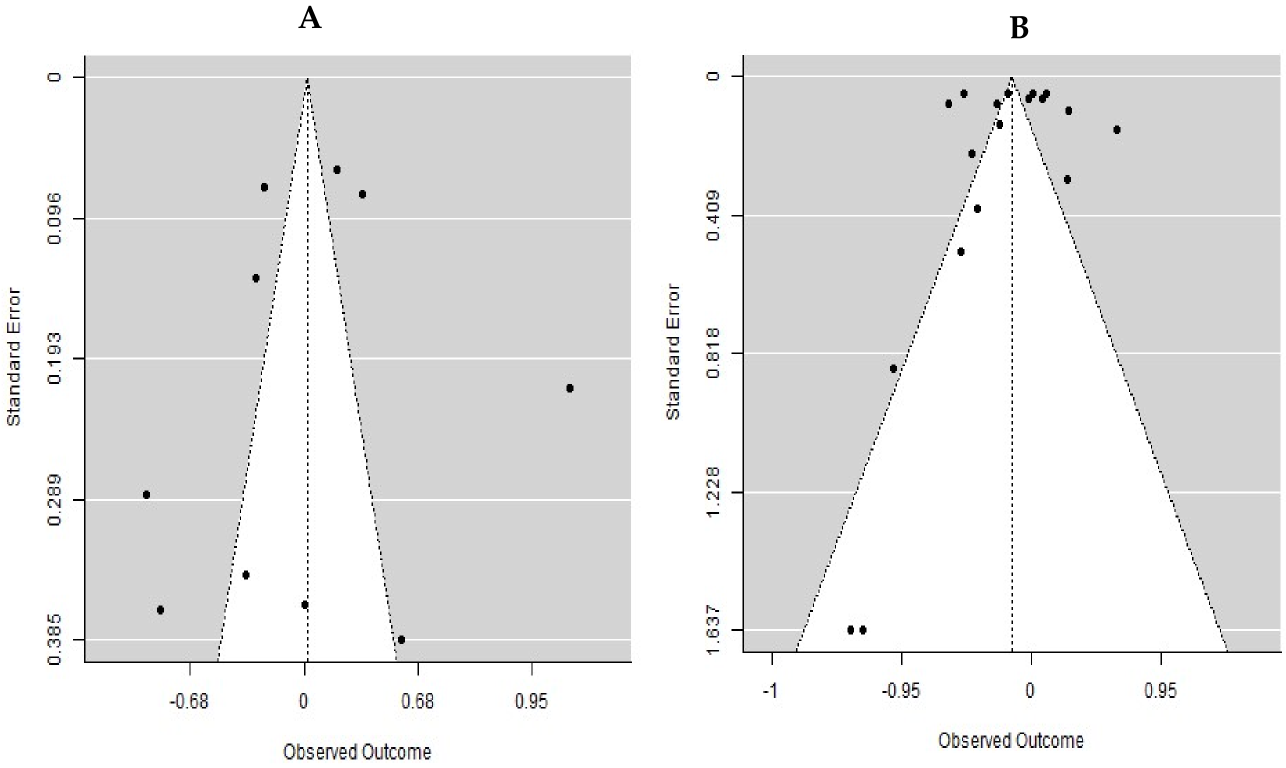

Supplementary Figure S3. A) Funnel Plot of Publication bias for a group of studies with intervention. **B)** Funnel Plot of Publication bias for a group Studies without Intervention.
